# Healing at sites prepared using different drilling protocols. An experimental study in the tibiae of sheep

**DOI:** 10.1371/journal.pone.0202957

**Published:** 2018-08-29

**Authors:** Vittorio Favero, Shigeru Sakuma, Karol Alí Apaza Alccayhuaman, Guillermo Alejandro Benedetto, Franco Bengazi, Daniele Botticelli

**Affiliations:** 1 Unit of Maxillo-Facial Surgery and Dentistry, University of Verona, Verona, Italy; 2 ARDEC Academy, Ariminum Odontologica, Rimini, Italy; 3 Facultad de Odontologia, Universidad de Buenos Aires, Buenos Aires, Argentina; 4 Faculty of Dentistry, University of Medical Science, La Habana, Cuba; University of Memphis, UNITED STATES

## Abstract

The aim of the experiment was to study the healing at implants installed in site prepared in bone type 1 using different rotation speeds and cooling strategies. The tibiae of twelve sheep were used as experimental sites. Two implant sites were prepared in each tibia using drills either at a high or a mixed speed under irrigation. At the mixed-speed sites, 60 rpm without irrigation were applied for the last drill, the countersink and during implant installation. Biopsies representing the healing after 1, 2, and 6 weeks were obtained and ground sections were prepared. At the histological analyses, after 1 week of healing, no new bone was found at both high- and mixed-speed sites. After 2 weeks of healing, small amounts of newly formed bone were observed in the cortical layer, reaching percentages of 3.6±3.0% at the mixed-speed sites, and of 2.2±1.5% at the high-speed sites. An irrelevant quantity of new bone was seen in the marrow compartments of a few specimens. After 6 weeks of healing, new bone was found in higher quantity, reaching in the cortical compartment 66.9±6.8% and 67.3±17.7% at the mixed- and high-speed sites, respectively. The respective percentages in the marrow compartment were 23.2±13.0% and 30.6±29.2%. No statistically significant differences between high- and mixed-speed groups were found. It was concluded that the use of the last drill and the installation of the implant with or without irrigation yielded similar bone healing and osseointegration.

## Introduction

Many factors related to the implant recipient site preparation may influence healing and osseointegration.[1] Aspects associated with the number of drills used, [2–5] their rotatory speed [6–9], diameters,[3,10] and conformation [3,11–16] have been widely investigated, as had the forces applied during site preparation,[17,18] the type of cooling applied [9,14,15,19–21] and the hardness of the bone, [3,22] which will affect heat production and healing processes.

The healing after preparation of osteotomies using low rotational speeds of 100, 500 and 1000 rpm [8] and high rotational speeds of 2,000, 30,000 and 400,000 rpm was also investigated [7] under abundant irrigation. It was concluded that the higher speeds minimized heat production and yielded a stronger biological response compared to the lower speeds.

The effect on the temperature of the coolants was also investigated. In bovine mandibles, osteotomies were prepared, and the temperatures were measured with thermoresistors at a depth of 3, 7 and 12 mm.[23] Without cooling, temperatures of about 51°C, 47°C, and 38°C were registered, respectively, while using saline as coolant a maximum temperature of 36–37°C were observed.

In an experiment in rabbits,[24] osteotomies were prepared in the tibiae with either low-speed drilling (50 rpm) without irrigation or high-speed drilling (800 rpm) with irrigation. It was concluded that bone healing was similar in both groups.

In an in vitro study,[15] different types of drills were used to prepare osteotomies in artificial bone cylinders that simulated type 1 bone. Traditional implant site preparation or site preparation for guided surgery were tested, with or without cooling, and using drilling speeds between 300–600 rpm. The temperature variations during site preparation were measured with a thermocouple. At the traditional site preparation, the use of the last drill without cooling produced an increase of temperature of about 12°C. In another in vitro study,[25] three drills with different geometry were used at a slow drilling speeds (50/150/300 rpm) without irrigation to prepare implant beds in type IV bovine bone disks. The temperature increases in values, but always below the threshold of 47°C.

In a randomized, controlled, parallel-group clinical trial, twenty-five patients were recruited.[26] Fifteen implants were installed in sites prepared using drills at high-speed drilling with irrigation (group A), while fifteen implants were installed in sites prepared using drills at low speed without irrigation (Group B). After 12 months, the success rate at an implant level was 93.3% in group A and 100% in group B. Marginal bone loss was similar in the two groups.

The heating produced by seating the implants into the recipient site was also evaluated.[27] Implants with different diameters were installed in the dense bone of the bovine mandibular ramus. A thermocouple was used to measure the increasing temperature within the bone close to the osteotomy before and during implant installation. It was shown that the smaller the diameter of the implants, the higher the increase in temperature induced by the friction of the implant to the bone surface of the osteotomy. The increasing temperatures registered were >0.1°C, >1°C, 1.4°C, and 8.6°C for the diameter of 5.7 mm 4.7 mm, 3.7 mm and 2.5 mm, respectively. These results were explained by the fact that, titanium being a much better heat conductor than bone, the larger surface of the wider implant could more effectively disperse the heat produced by the friction of the implant compared to smaller implants. However, this experiment was performed in non-vital bone so, clinically, it may have an irrelevant effect on osseointegration.

This evidence showed the importance of the irrigation and of the use of a low drilling speed to reduce the increase of temperature in the bone. However, an increase of temperature was also detected during the use of the last drill and during implant installation. Clinicians might need more information about bone healing and implant osseointegration when a low speed without irrigation is applied during the latest procedures of osteotomy preparation and implant installation.

Hence, the aim of the present experiment was to study the healing at implants installed in sites prepared in bone type 1 using different rotation speeds and cooling strategies.

## Material and methods

The rules for animal care requested by the local authorities were strictly followed. With this aim, the experiment was performed in strict accordance with the recommendations included in the Code of Good Practice of the Laboratory of the CETEX (Centro de Toxicología Experimental, Cuba) and the Code for the use of laboratory animals of the CENPALAB (Centro Nacional para la Producción de Animales de Laboratorio, Cuba). The protocol was approved by the Ethical Committee for the Investigation of the Faculty of Stomatology of the University of Medical Science of La Habana, Cuba (#012/2013, approved on November 20, 2013). All surgeries were performed under anesthesia and under strict control of veterinaries. All efforts were made to minimize suffering of the animals.

### Sample and location for animal treatment and maintenance

Twelve female Pelibuey sheep, with mean age of about 3 years and a mean weight of about 34 Kg were selected at the CENPALAB. All surgeries and euthanasia were carried out between February and June 2014 at the Center of Experimental Surgery (CENCEX), Faculty of Medicine Victoria de Girón, University of Medical Science of La Habana, Cuba. After the surgery, all animals were maintained at CENPALAB.

### Randomization and allocation concealment

The ARRIVE checklist was adopted as guide (S1 Checklist). To eliminate interference among animals, a split-mouth design was used. According to the Three R requirements, [28] this design allowed the use of a small number of animals to describe the event of healing with sufficient approximation. An experimental study was taken into consideration, in which a statistically significant lower bone-to-implant contact and bone density was found in implants placed in the mandible of five sheep without cooling compared to cooling administrated using different types of irrigation (external and/ or external).[29] a difference of 16% with a standard deviation of about 10%. Considering these values clinically relevant, 5 pairs of subjects were calculated to be able to reject the null hypothesis that this response difference is zero with a power 0.8 and α 0.05. An n = 6 was used to increase the power. Sheep tibiae were selected because of the extreme hardness that may have stressed the challenge from heating and a width of the cortical bone (~4 mm), that may have guaranteed a sufficient primary stability of the implants.

**Fig 1:**
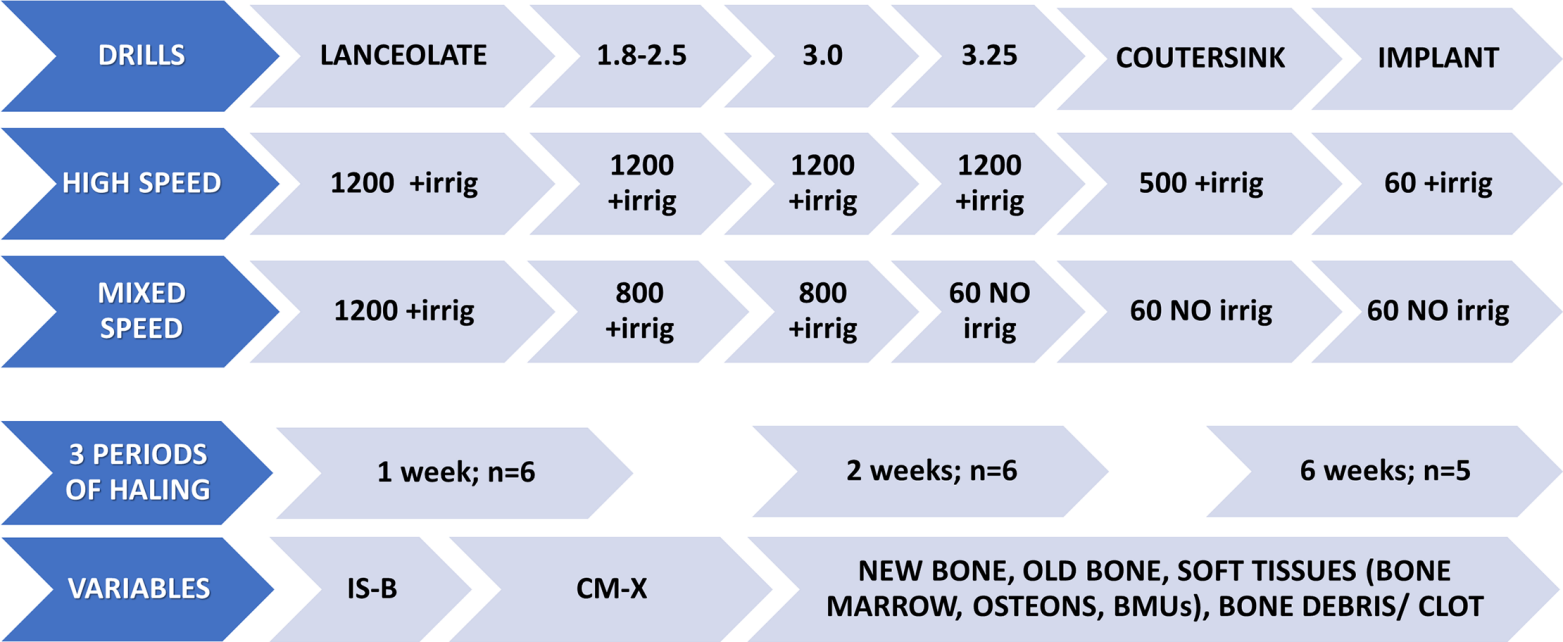
Diagram illustrating the experiment design. Drills diameters expressed in millimeters (mm). Speed expressed in round per minute (rpm). + irrig = irrigation provided; NO irrig = no irrigation provided; IS = implant shoulder; B = CM = limit between the cortical layer and the marrow compartment; X = most apical contact of the bone to the implant surface.

Two different procedures for implant recipient sites preparation were performed (Fig 1), one prepared with the drills rotating at a higher speed (high-speed sites) and the other prepared at a lower speed (mixed-speed sites).

The randomization was performed electronically (www.randomization.com) by an author not involved in any surgical procedures (DB). Two groups were formed, each composed of six animals. In the first group, the tibiae of both side were used, while in the second only the right side.

The surgeon (FB) was informed of the treatment after having exposed the experimental site.

No information about the location of test and control sites or period of healing were provided to the examiner at the histological analysis. The examiner (VF) was carefully trained before the measurements.

### Anesthetic procedures

The animals fasted for 24 hours preoperatively, but were allowed to drink water ad libitum. Anesthesia was performed by veterinarians, who administered 0.2mg/Kg of midazolam (Dormicum®, Roche, Basel, Switzerland) and 5mg/kg of ketamine (Ketamina-50, Liorad, La Habana, Cuba). After orotracheal intubation, the anesthesia was kept with 3% isofluorane (Isofluorane-vet, Merial, Toulouse, France) mixed with 5l/min oxygen. Mepivacaine HCI 2% with 1:100,000 epinephrine was provided locally.

### Surgical procedures

All surgeries were performed under good clinical and laboratory practices. The legs of the sheep were shaved and then disinfected with chlorhexidine digluconate. After that, a 15cm long incision was performed in the leg, corresponding to the ventral aspect of the tibia. Skin, muscular fascia and periosteum were dissected and the bone surface of the tibia denuded.

**Fig 2:**
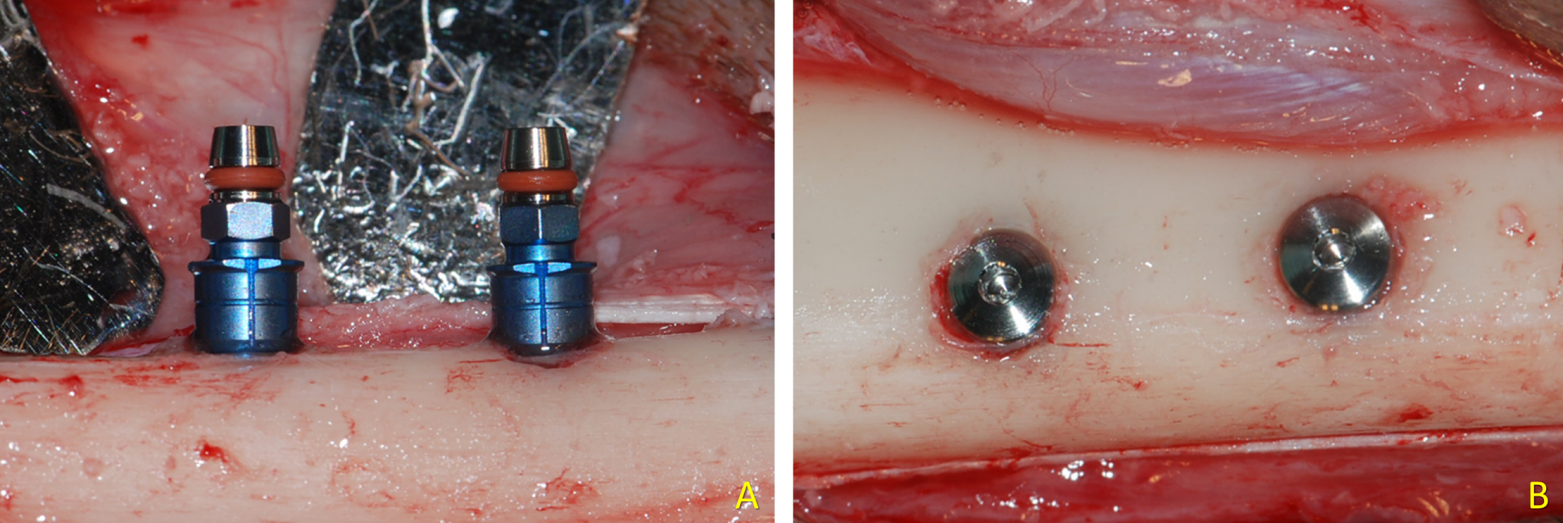
Implants installed in the tibia. (A) lateral view. Mounters still in situ. (B) Occlusal view. Cover screws were placed on the top of the implants.

Two experimental sites were selected, and drills of increasing diameters were used in the same sequence to prepare both high- and mixed-speed sites. Different rounds per minutes and cooling procedures with saline were used, adopting a different protocol from that suggested by the implant company. Two cylindrical screw-shaped not self-tapping implants BTI Interna® (Biotechnology Institute S.L., Miñano, Alava, Spain), 7.5mm long and 3.5mm in diameter, were installed at 60 rpm, without cooling at the mixed-speed side and with cooling at the high-speed side. No tapping procedure were applied prior installation. The shoulder of the implants was placed about flush to the top of the cortical bone crest and protected with cover screws (Fig 2A and 2B). The wounds were closed with resorbable sutures.

After one week, the surgery was repeated in the contralateral limb of the animals of the first group.

### Maintenance after surgery

The day of the surgery, Gentamicin® 8ml/100kg every 12 hours was administered and Metamizole sodium monohydrate 500 mg/ml, 1ml/10 Kg i.m. The doses of both drugs were repeated every 24 hours for the following 3 days. To reduce the distress after surgery, the sheep were kept in the animal house, individually, in a roofed shed, with a concrete floor. The boxes were cleaned daily and the animals had free access to water. The diet was based on balanced food made of cereals, protein, and concentrates of vitamins and minerals. Green forages were added. The wounds were disinfected by the veterinaries of the center every day during the first week and, subsequently, inspected three times per weeks for clinical signs of complications for the full duration of the experiment.

### Euthanasia

The animals were first anesthetized and then euthanized with an overdose of pentobarbital sodium. The animals of the first group were euthanized after 1 week of the second surgery so that biopsies representing the healing after 1 and 2 weeks were obtained. The animals of the second group were euthanized after 6 weeks. The tibia were retrieved, trimmed and immersed in formalin solution.

### Histologic preparation

The histological slides were processed in the laboratory of histology of the Faculty of Odontology, University of La Habana, Cuba. Block sections, each containing one implant, were prepared and dehydrated in a series of graded ethanol. After infiltration in resin (Technovit® 7200 VLC, Kulzer, Friedrichsdorf, Germany), a cut was carried out through the center of the implants, following its long axis and in a transverse plane with respect to the tibia. A precision slicing machine was used for this (Exakt®, Apparatebau, Norderstedt, Germany). A slice of about 150–200μm was prepared and reduced to a thickness of about 60μm with a cutting–grinding device (Exakt®, Apparatebau, Norderstedt, Germany). The histological slides were subsequently stained with Stevenel’s blue and alizarin red.

### Histological examination

An Eclipse C*i* microscope (Nikon Corporation, Tokyo, Japan), equipped with a digital video camera (Digital Sight DS-2Mv, Nikon Corporation, Tokyo, Japan) connected to a computer was used for histological examination. The software NIS-Elements D (Laboratory Imaging, Nikon Corporation, Tokyo, Japan) was adopted for measurements.

**Fig 3:**
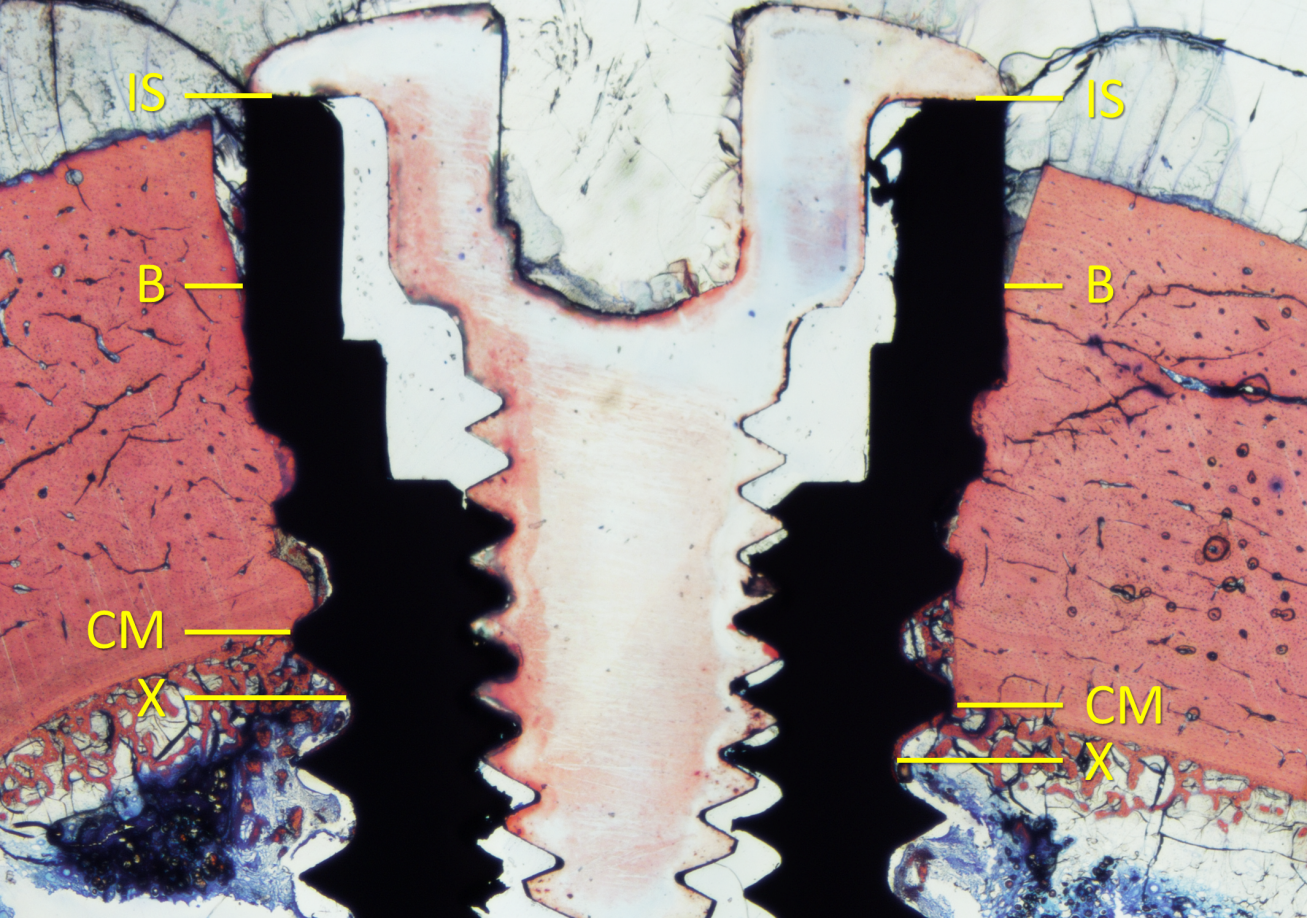
Diagram illustrating the landmarks for the histological evaluation. (IS) the shoulder of the implant, (B) the most coronal bone-to-implant contact, (CM) the limit between the cortical layer and the marrow compartment, (X) the most apical bone-to-implant contact.

The following landmarks were identified (Fig 3): (IS) the shoulder of the implant, (B) the most coronal bone-to-implant contact, (CM) the limit between the cortical layer and the marrow compartment, (X) the most apical contact of the bone to the implant surface. Moreover, (A) the apex of the implant was also identified.

The linear distances between IS and B (IS-B) and CM and X (CM-X) were measured parallel to the long axis of the implant at both sides of the implant at a magnification of x100. Moreover, the amount of new bone, old bone, total bone (new + old bone), soft tissues (marrow spaces, osteons, BMUs, provisional matrix), and other tissues (bone debris, bone particles and clot) in contact with the implant surface were measured at a magnification of x200, between B and C/M (cortical layer). New mineralized bone-to-implant contact was also measured between C/M and A (marrow compartment). Percentages in relation to the total implant regions evaluated were subsequently calculated. All measures were performed at both sides of the implants and mean values were obtained of the two measures for each variable. The interception point,^26^ that is the point at which in a graph the two proportional lines of new and old bone on the implant surface intersect with each other, was also calculated for both groups and defined as time of occurrence (days) and percentage of osseointegration reached (%).

### Statistical analysis

Mean values, standard deviation (SD) and lower-upper 95% confidence intervals (CI) of the differences between high- and low-speed sites were calculated. The distance CM-X and the new bone in contact with the implant surface were the primary variables.

Differences between high- and low- speed were analysed using Wilcoxon signed rank test included in the SPSS Statistics 19 (IBM Inc., Chicago, IL, USA). The level of significance was set at α = 0.05.

## Results

**Fig 4:**
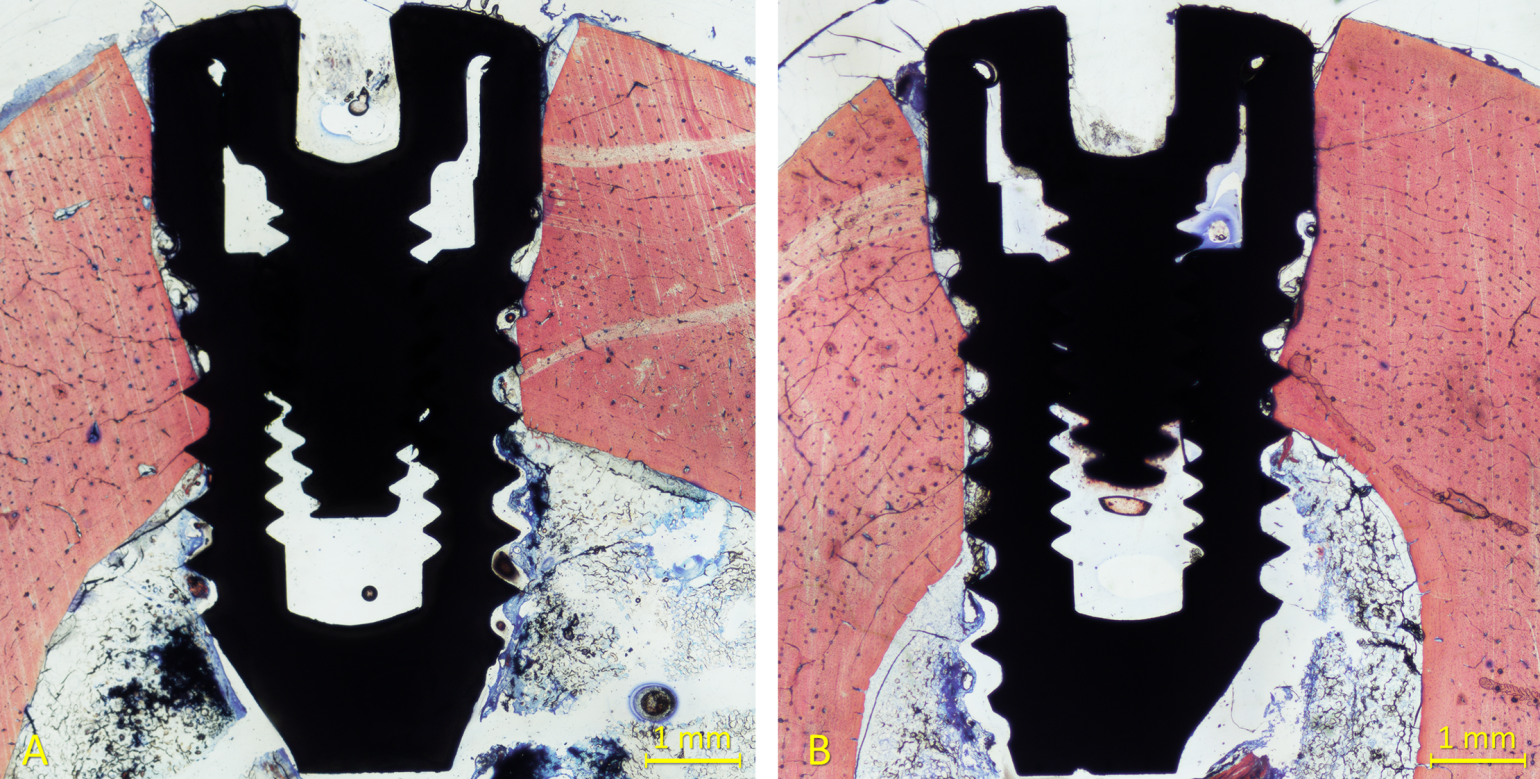
Ground sections representing the healing after 1 week at the (A) high- and (B) mixed-speed sites. Incongruences were observed between the osteotomies and the implants. No new bone was detected at this time of healing. Stevenel’s blue and alizarin red stain.

**Fig 5:**
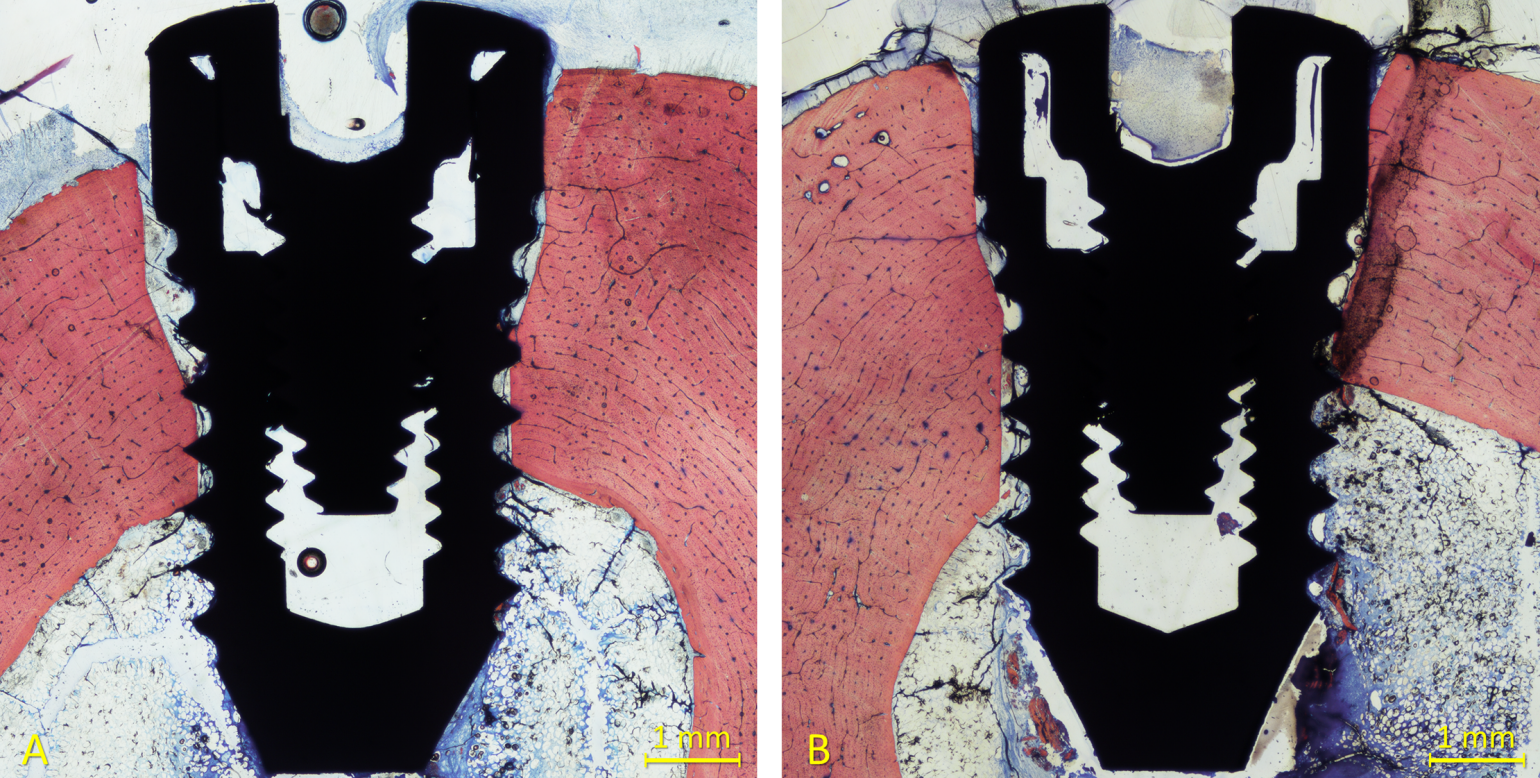
Ground sections representing the healing after 2 weeks at the (A) high- and (B) mixed-speed sites. Incongruences between the osteotomies and the implants were still present. Little amounts of new bone were observed. Stevenel’s blue and alizarin red stain.

**Fig 6:**
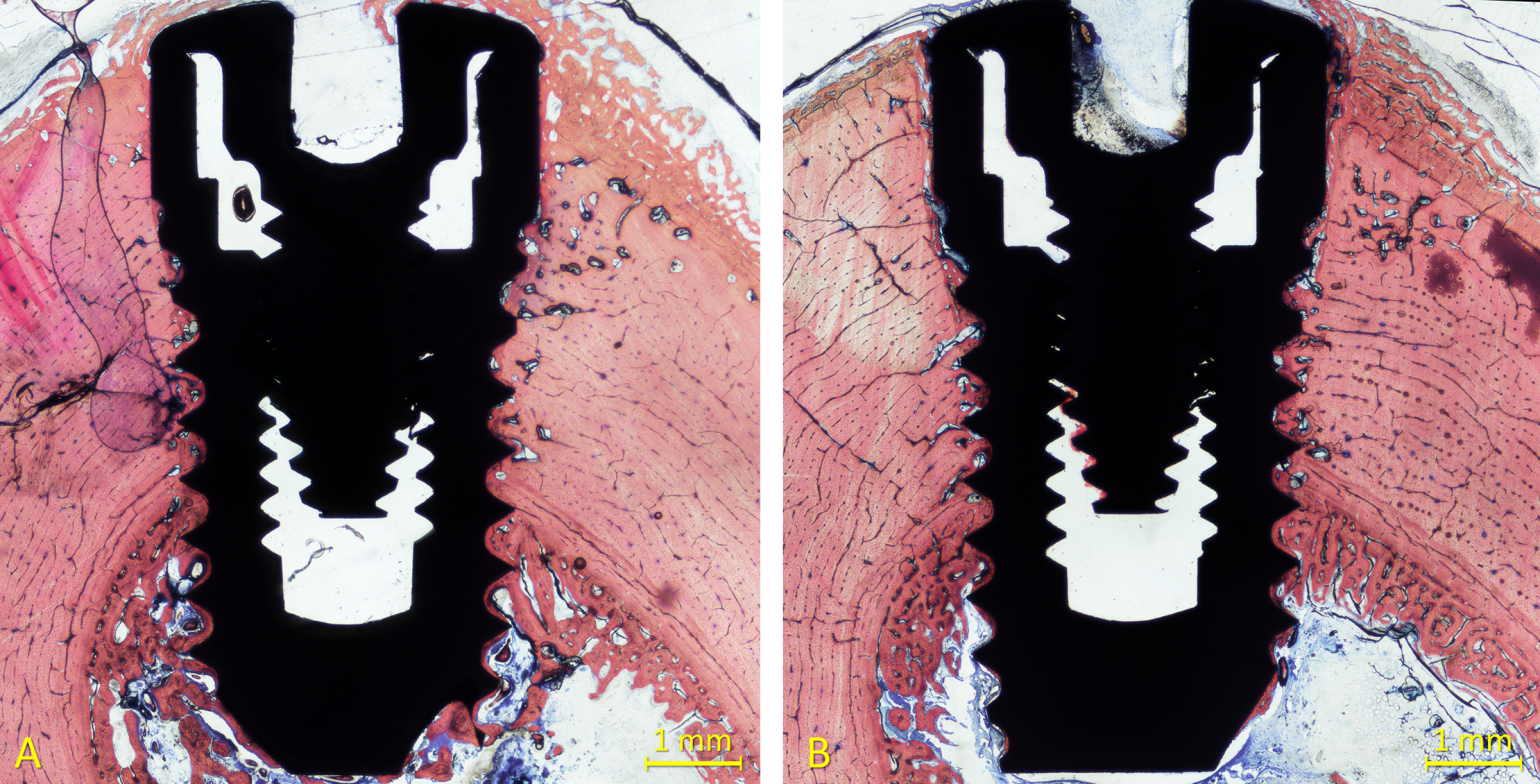
Ground sections representing the healing after 6 weeks at the (A) high- and (B) mixed-speed sites. The incongruences between the osteotomies and the implants were filled with newly formed bone. New bone was also formed on the surface of the implant protruding within the bone marrow compartment. Stevenel’s blue and alizarin red stain.

All implants were available for histological preparation. However, both implants of one sheep of the 6-week healing group were not integrated so that an n = 5 was obtained. For both 1- and 2-week healing groups, an n = 6 was achieved. Ground sections representing the healing at the high- and mixed-speed sites are illustrated in Fig 4 (1 week), Fig 5 (2 weeks) and Fig 6 (6 weeks).

### Levels of osseointegration

After 1 week of healing, the distance IS-B was 2.2±1.3 mm and 1.8±1.1 mm at the high- and mixed-speed sites, respectively (Table 1). No resorptive processes or new bone apposition were seen at this stage of healing, so the bone crest was still about at the same level of the implant shoulder. A discrepancy between the recipient bed and the implant body explain values of ~2 mm of the distance IS-B. After 2 weeks of healing, the distances IS-B were similar to those of the previous period of healing, being 2.2±0.6 mm and 2.0±0.7 mm at high- and low-speed sites, respectively. After 6 weeks of healing, the distance IS-B was close to 0 mm at both sites.

**Table 1:** Levels of osseointegration.

	HIGH–SPEED		MIXED—SPEED		Difference of the means	
	IS-B	CM-X	IS-B	CM-X	IS-B	CM-X
1 week (n = 6)	2.2±1.3	0.0±0.0	1.8±1.1	0.0±0.0	0.3±0.9; C.I. -0.4; 1.1	0.0 ±0.0; C.I. 0.0; 0.0
2 weeks (n = 6)	2.2±0.6	0.2±0.4	2.0±0.7	0.1±0.2	0.2±0.5; C.I. -0.2; 0.6	0.1±0.2; C.I. -0.1; 0.3
6 weeks (n = 5)	0.0±0.0	1.6±0.8	0.1±0.1	1.5±0.6	-0.1 ±0.1; C.I. -0.1; 0.0	0.1 ±0.9; C.I. -0.7; 0.9

Mean values ± standard deviations and confidence interval of the difference of the mean (C.I. lower; upper 95%). Values in millimeters. IS = coronal margin of the implant; B = coronal level of osseointegration; CM = limit between the cortical layer and the marrow compartment; X = most apical bone-to-implant contact. p<0.05 between high and mixed speed.

After one week, no new bone was seen forming on the implant surface within the marrow compartment (CM-X = 0 mm). After two weeks, very small amounts of bone (CM-X = 0.1–0.2 mm) were seen in few specimens. After 6 weeks, new bone formation was evident and CM-X increases to 1.6±0.8 mm and 1.5±0.6 mm at the high- and mixed-speed, respectively. No statistically significant differences were found between mixed- and high-speed sites for any of the variables analyzed at any of the periods of healing.

### Quality of osseointegration

**Fig 7:**
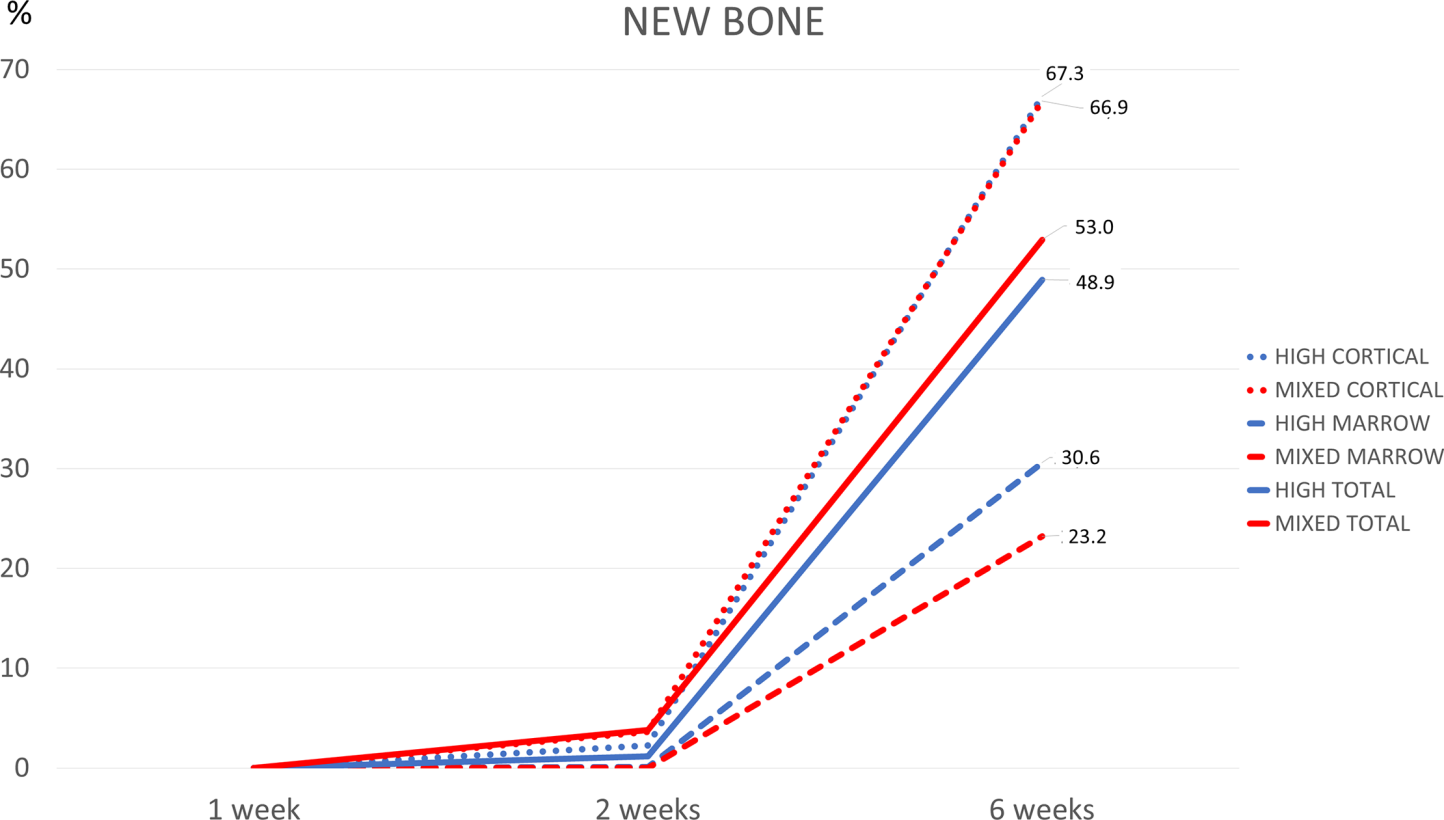
Graph representing the amount of new bone in the high- (HIGH) and mixed- (MIXED) speed groups, in the various compartments (cortical, marrow and both) after 1, 2 and 6 weeks of healing.

After 1 week of healing, (Tables 2 and 3, Fig 7) no new bone was found on the implant surface at either the cortical or the marrow compartments.

**Table 2:** Tissues in contact with the implant surface in percentage in the cortical compartment.

		**New bone**	**Old bone**	**Soft tissue**	**Debris/ clot**
**HIGH–SPEED**	1 week (n = 6)	0.0±0.0	13.0±3.7	80.4±8.2	6.6±6.1
	2 weeks (n = 6)	2.2±1.5	14.9±3.0	81.2±2.9	1.7±1.1
	6 weeks (n = 5)	67.3±17.7	9.5±3.7	23.2±15.8	0.0±0.0
**LOW–SPEED**	1 week (n = 6)	0.0±0.0	13.0±4.5	83.0±5.3	4.1±4.3
	2 weeks (n = 6)	3.6±3.0	12.4±5.8	82.8±5.2	1.1±2.1
	6 weeks (n = 5)	66.9±6.8	8.0±3.3	25.2±10.0	0.0±0.0

Means ± standard deviations.

p<0.05 between high and mixed speed.

**Table 3:** Tissues in contact with the implant surface in percentage in the marrow and in both compartments.

		**Marrow compartment**		**Both compartments**	
		New bone	Old bone	New bone	Old bone
**HIGH–SPEED**	1 week (n = 6)	0.0 ±0.0	0.0±0.0	0.0 ±0.0	6.5±1.8
	2 weeks (n = 6)	0.1±0.2	0.0±0.0	1.2±0.7	7.4±1.5
	6 weeks (n = 5)	30.6±29.2	0.0±0.0	48.9±19.8	4.7±1.8
**LOW–SPEED**	1 week (n = 6)	0.0±0.0	0.0±0.0	0.0±0.0	6.5±2.2
	2 weeks (n = 6)	0.0±0.0	0.0±0.0	1.8±1.5	6.2±2.9
	6 weeks (n = 5)	23.2±13.0	0.0±0.0	45.0±6.9	4.0±1.6

Means ± standard deviations.

p<0.05 between high and mixed speed.

Old bone was found at percentages of 13% in the cortical region at both the high- and mixed-speed sites. Soft tissues were the most represented, occupying the spaces within the threads and the discrepancy between the recipient site and the implant surface. The soft tissues often contained bone debris/ particles and clot remnants in contact with the implant surface at both groups.

**Fig 8:**
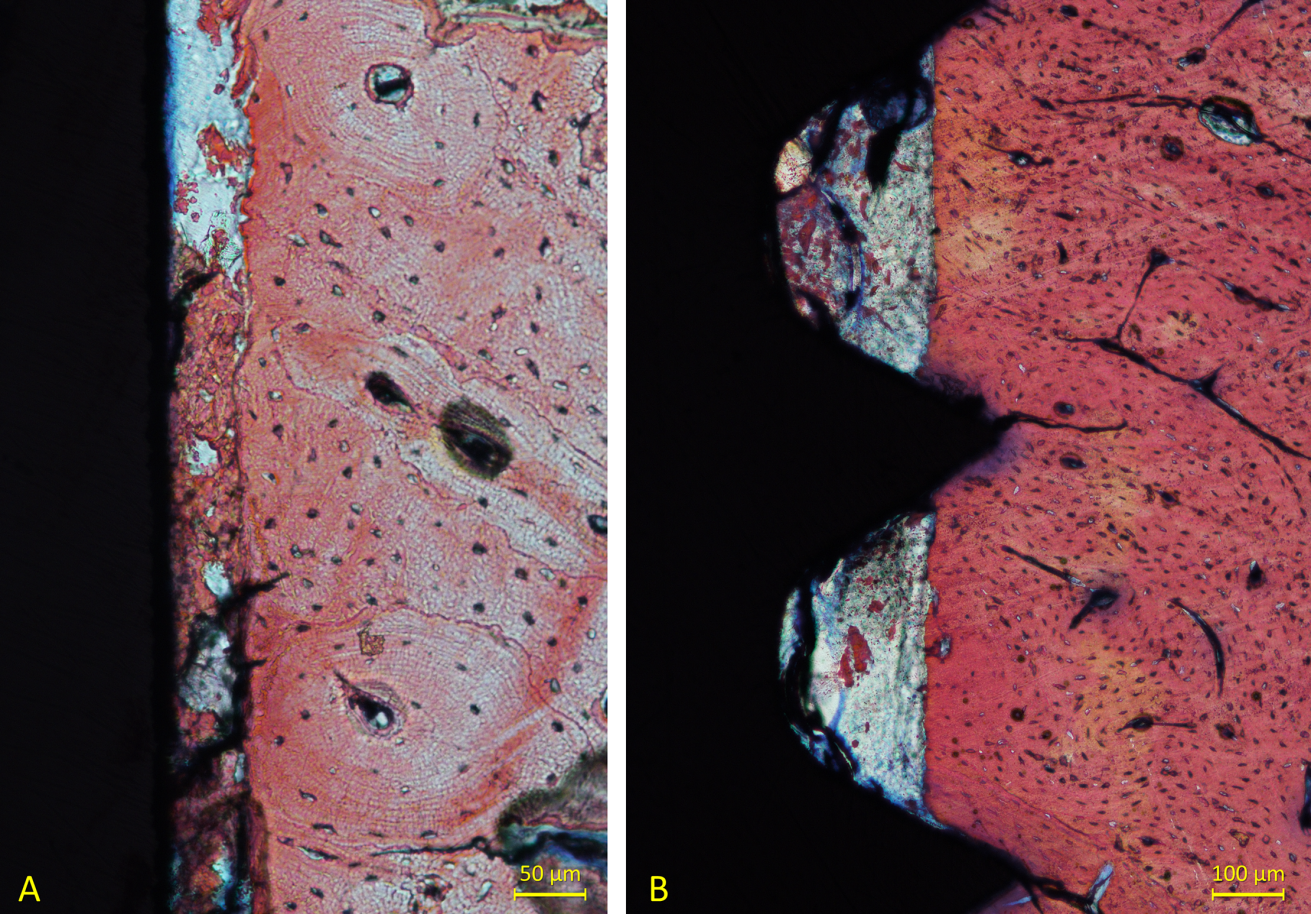
(A) Bone particles mixed to clot residues located between the osteotomy and the implant surface after 1 week of healing. Stevenel’s blue and alizarin red stain. (B) Bone particles within the threads of the implants after 2 weeks of healing. Stevenel’s blue and alizarin red stain.

After 2 weeks of healing, small amounts of newly formed bone were observed in the cortical layer, reaching percentages of 2.2±1.5% at the high-speed sites and of 3.6±3.0% at the mixed-speed sites. A very low amount of new bone was seen in the marrow compartments, but this was observed only in a few specimens. Old bone was still present in similar quantity compared to the previous period of healing, revealing that low resorptive processes occurred during the first 2 weeks of healing. Very little amounts of debris and particles were found at this interval of healing (Fig 8A and 8B).

**Fig 9:**
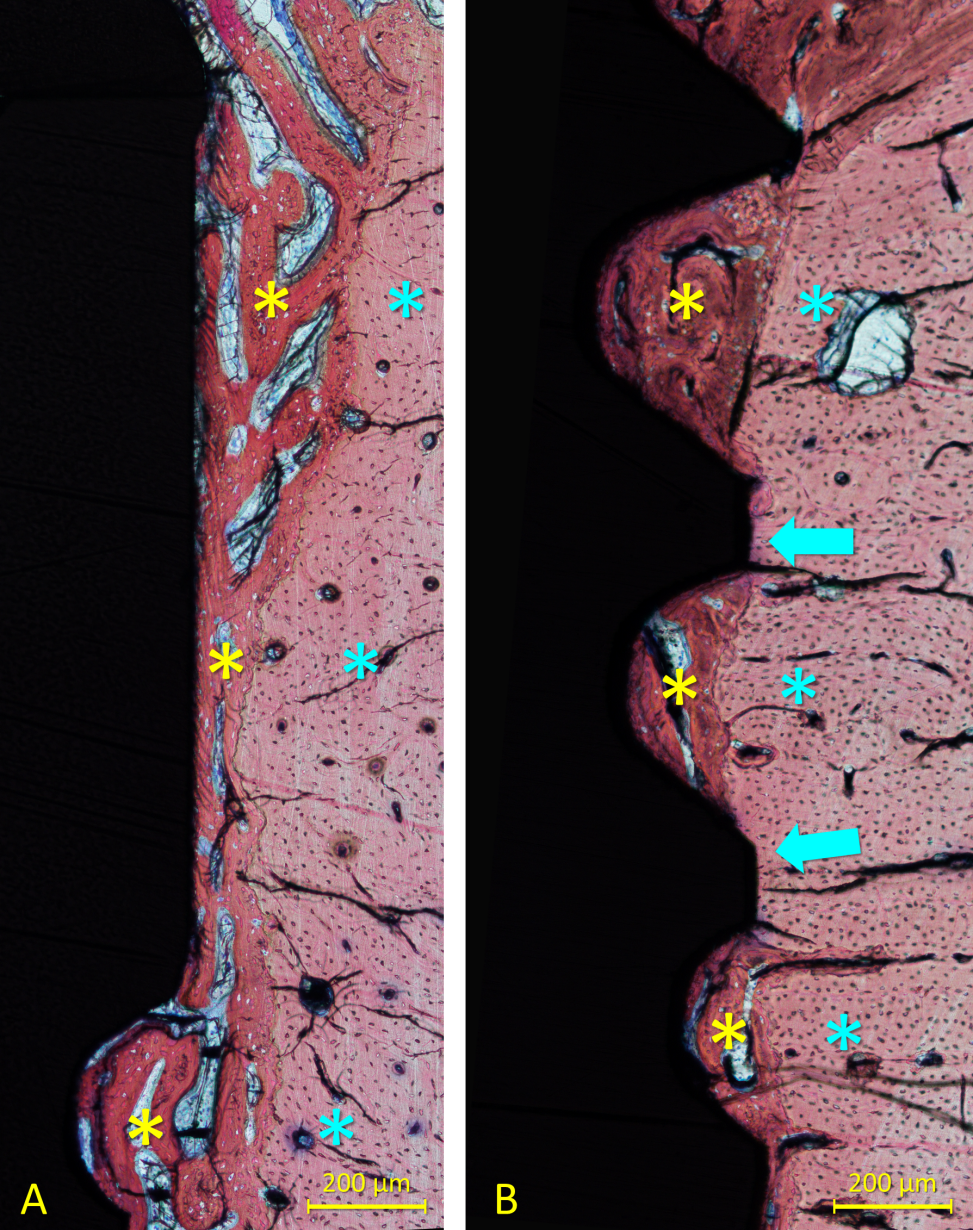
Ground illustrating the healing after 6 weeks at the high speed sites. (A) New bone (yellow stars) filled the incongruences between the implant surface and the old bone (light blue stars). (B) New bone filled the spaces between threads. Old bone was found in contact with some regions of the implant surface (light blue arrows). Stevenel’s blue and alizarin red stain.

After 6 weeks of healing, new bone was found in higher quantity, in the cortical compartment reaching 67.3±17.7% and 66.9±6.8% at high- and mixed-speed sites, respectively. The discrepancies between the osteotomies and the implants that were identified after 1 and 2 weeks of healing were found filled with newly formed bone after 6 weeks of healing (Fig 9A).

**Fig 10:**
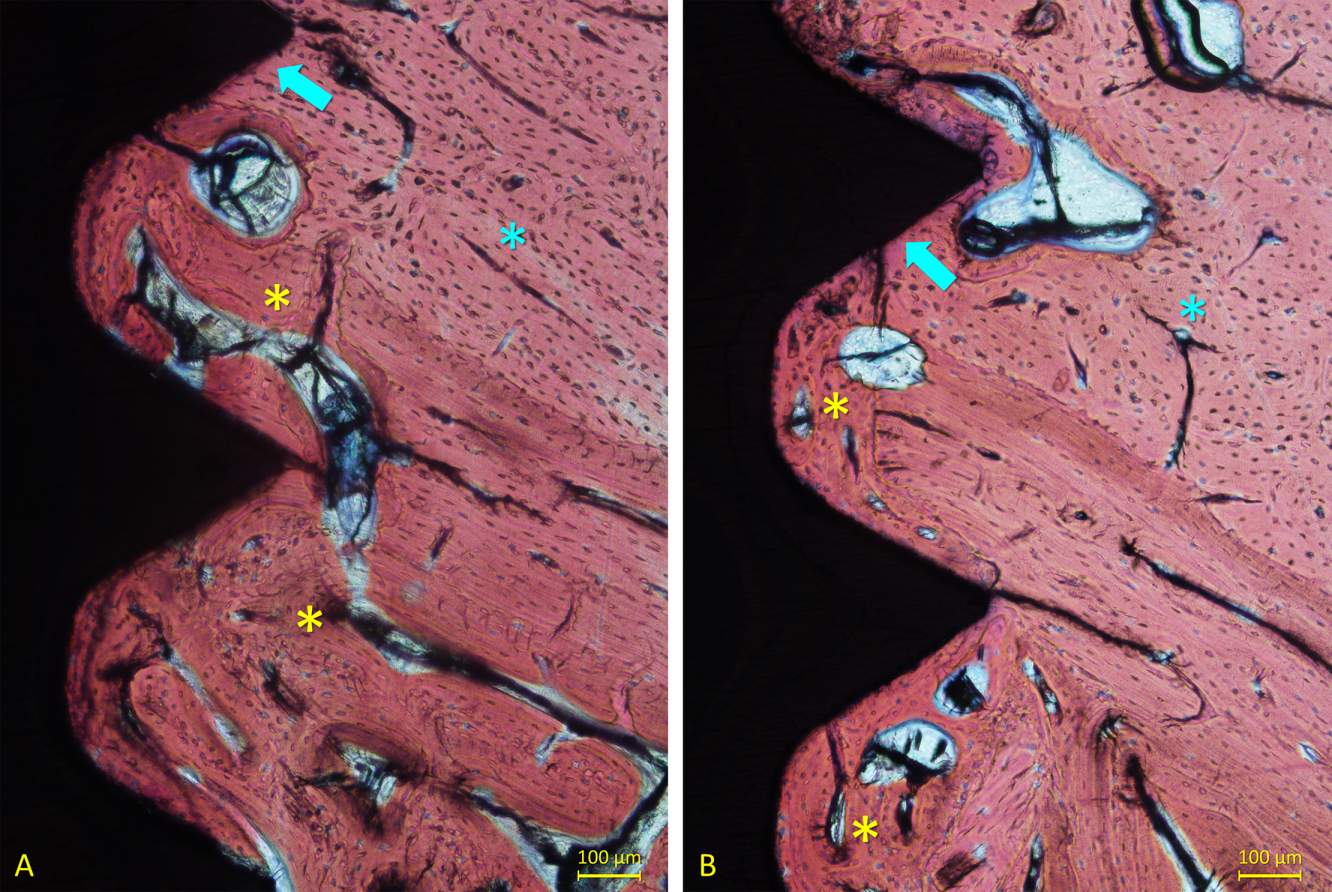
Ground illustrating the healing after 6 weeks at the mixed speed sites. New bone (yellow stars) filled the spaces between threads. Old bone (light blue stars) was occupying regions around the implant and in some instances in contact with the surface (light blue arrow). Stevenel’s blue and alizarin red stain.

The spaces between threads were also filled with new bone. Old bone was observed at percentages of 9.5±3.7% and 8.0±3.3% at the high- and mixed-speed sites, respectively. In some instances, old bone was still in contact with the implant surface (Fig 9B; Fig 10A and 10B).

In the marrow compartment, new bone in contact with the implant surface was 30.6±29.2% and 23.2±13.0% at the high- and mixed-speed, respectively. Considering both compartments, the total amount of newly formed bone was 48.9±19.8% and 45.0±6.9% at the high- and mixed-speed sites, respectively. No statistically significant differences were disclosed between high- and mixed-speed sites for any variable analyzed at any of the periods of healing. The difference of the means ± standard deviations and confidence interval (CI lower; upper 95%) in the cortical and marrow compartments after 6 weeks of healing are reported in Table 4.

**Table 4:** Difference of the means ± standard deviations and confidence interval in the cortical and marrow compartments after 6 weeks of healing.

	**Cortical compartment**	**Marrow compartment**	**Both compartments**
**New bone**	0.4±17.4 C.I. -13.5; 14.4	7.4±17.5 C.I. -6.6; 21.4	3.9±14.1 C.I. -7.4; 15.2
**Old bone**	1.5±6.3 C.I. -3.6; 6.5	0.0±0.0 C.I. 0.0; 0.0	0.7±3.2 C.I. -1.8; 3.3

C.I. lower; upper 95%

Values in percentages (%).

**Fig 11:**
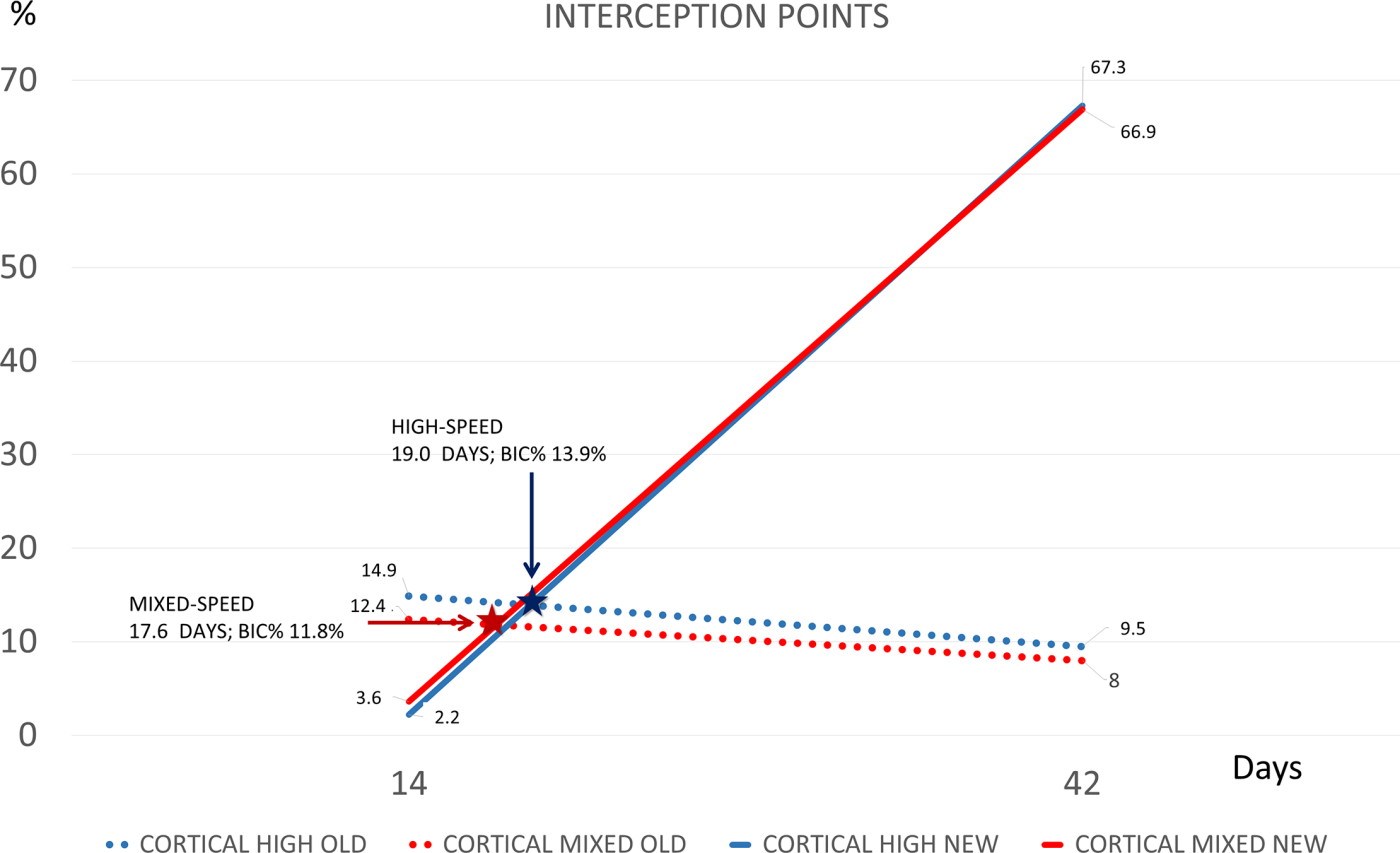
Graph representing the interception point, defined as the point at which the two proportional lines of new and old bone on the implant surface intersect with each other. It is expressed as time of occurrence (days) and percentage of osseointegration reached (%). The blue and the red stars indicate the interception points for the high- and the mixed-speed groups, respectively.

The interception point occurred after 19 days at 13.9% of osseointegration in the high-speed group and after 17.6 days and at 11.8% of osseointegration in the mixed-speed groups (Fig 11). When also the marrow compartment was considered, the respective data were 17.4 days at 7.1% in the high-speed group and 16.7 days at 6.0% in the mixed-speed group.

## Discussion

In the present experiment, the healing at three different periods at implants installed in sites either prepared with drills at high or at a mixed speed. Only two implants were found not integrated in the group 6-week of healing. No differences were seen in new bone formation in both cortical and bone marrow compartment.

Several studies have reported data on implant sites preparation using drills at different speeds, and showed that, if a good irrigation is provided, a good integration may be achieved. In an experimental study evaluating the effect of drilling speed on early bone healing [8], thirty-six implants were placed in the proximal tibiae of 6 beagles. The drilling speeds used were100, 500, and 1000 rpm under irrigation. The healing was studied after 1 and 3 weeks from implant installation. The implants were well integrated independently from the speed drilling, without statistically significant differences in bone-to-implant contact among groups. However, it was concluded that the strongest biological response was seen at the sites prepared at 1000 rpm.

In another study in rabbits tibia, [6,7] speeds of 2,000 rpm, 30,000 rpm, and 400,000 rpm were applied to prepared osteotomy, and cooling was added. A negative relationship was found between drill speed and production of heat.[6] Moreover, the healing investigated after 2, 4 ad 6 weeks revealed that highest speed preparation resulted with the greatest degree of healing.[7]

These results agree with those of the present study, in which small differences in new bone formation in favor of the high speed were seen compared to the mixed speed, even though not statistically significant. However, these differences were more evident in the marrow compartment compared to the cortical layer.

In another study performed on ten freshly dissected sheep mandibles, [21] cooling against no cooling was tested by studying the effects of the amount of irrigation on heat generated during implant site preparation. Osteotomies were prepared using four consecutive drills. A thermocouple was inserted into the bone in proximity of the osteotomy, and the temperature measurements were performed immediately before and after the preparation of the sites. Three different procedures were carried out, one without cooling, and two with irrigation, using 12 ml/min and 30 ml/min of saline volume, respectively. The temperatures in the unirrigated group were significantly higher than the other two groups, while no statistically significant differences were found between the two groups with irrigation.

In the present experiment, at the mixed-speed sites, the last drill, the countersink procedure and the subsequent implant installation were performed at 60 rpm with no irrigation. At the high-speed sites, the last drill was used at 1200 rpm, the countersink procedure at 500 rpm, and the implant was finally installed at 60 rpm, all procedures performed under conspicuous irrigation. As reported before, no differences were found between mixed- and high-speed sites, suggesting that both procedures resulted in similar outcomes regarding osseointegration and parent bone resorption. This, in turn, means that the last procedures to prepare the recipient site, even when performed in a hard bone such as that of the tibia, may be performed without cooling, providing that a low speed (~60 rpm) is used.

The results from the present experiment agree with those from another similar study in the sheep tibia [30] in which implants were installed in sites where yellow marrow was either removed or left in place. The results were evaluated after 1 and 3 months of healing. After 1 month, similar amounts of osseointegration to those reported in the present study were found. Additionally, the present study showed that bone formation started after 2 weeks, even though at a very low rate. Considering together the results from these two studies, it appears that most of bone apposition occurs between 2 and 4 weeks of healing at implants installed in the sheep’s tibia. Other experimental models presented a different rate of bone formation. For instance, in an experiment in rabbits, implants were installed in the tibia.[31] After five days of healing, a high degree of new bone in contact with the implant surface was found (~20%).

In the present study, the cortical bone found in the tibiae was very hard and could be classified as Type I [32], hardness similar to that of the mandibular symphysis or in the distal segments of an atrophic mandible in human. After 6 weeks of healing, in the cortical compartment, old bone in contact with the implant surface was still present in percentage of 8–9.5%. These outcomes are comparable to those reported in an experiment in dogs in which cortical and marrow compartments were studied. In the cortical region, after 30 days of healing, 15.6% of old bone was found in contact with the implant surface [33]. Phenomena of reabsorption of old bone and new bone apposition carried out by BMUs (Basic Multicellular Units) appeared to occur over time, at a distance from the implant surface that generally does not exceed 200 micron.[34]

A recent article described the influence of different variables on osseointegration.[35] It illustrated the degree of influence on osseointegration using a new parameter, called interception point, defined as the point at which the two lines of a graph expressing old bone resorption and new bone apposition onto an implant surface intersect with each other. It was defined as time of occurrence (in days) and percentage of contact of old or new bone to the implant surface. These parameters showed a faster rate of bone apposition in the rabbits, followed by dogs and then humans. A slower osseointegration was seen in the cortical bone compared to the spongiosa.[33] The modification of the surfaces was also shown to play an important role.[36–40]

New bone formation on the implant surface located within the marrow compartment appeared to start from the parent bone of the cortical layer and spread towards the apical portion of the implant. This agrees with other experimental studies that showed that bone was forming from the parent basal bone from which osseointegration could proceed to cover the surface of the implant.[41–44]

In an article previously mentioned the progression of the osseointegration from the parent cortical bone of the tibia towards the apex of the implants was also evaluated.[30] No further apical progression of osseointegration was found between 1 and 3 months of healing. The present article showed that the apical progression of osseointegration occurred between 2 and 6 weeks. Again, considering together the results from these two studies, it may be suggested that bone apposition in an apical direction occurs between 2 and 4 weeks of healing at implants installed in the sheep’s tibia.

The margin of the implants were placed at about the level of the top of the cortical bone. After one and two weeks of healing, the first contact of bone to the implant was located at ~2 mm from the implant margin. This may be explained by the discrepancy between the osteotomy and the implant surface and by the very low rate of bone formation during the first two weeks of healing. However, after further 4 weeks, bone formation increased to such a level as to fill the discrepancy completely.

## Conclusions

In conclusion, the use of the last drill and the installation of the implant in a bone type 1 with or without irrigation yielded similar bone healing and osseointegration.

It has to be kept in mind that the present study reported data from an animal experiment, and any inference to clinical situation in human should be carefully estimated. Nevertheless, the present experiment tested extreme conditions in relation of the hardness of the bone. This, in turs, means that similar results may be expected in clinical situations, especially in the presence of a more favorable density of bone.

## Supporting information

S1 Checklist (DOCX)
