# Supplementary material for: Healing at sites prepared using different drilling protocols. An experimental study in the tibiae of sheep
Source: PLoS One. 2018 Aug 29;13(8):e0202957. doi: 10.1371/journal.pone.0202957 (PMC6114797; doi:10.1371/journal.pone.0202957)

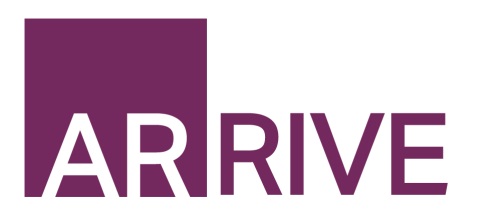


The ARRIVE Guidelines Checklist

Animal Research: Reporting In Vivo Experiments

Carol Kilkenny^1^, William J Browne^2^, Innes C Cuthill^3^, Michael Emerson^4^ and Douglas G Altman^5^

*^1^The National Centre for the Replacement, Refinement and Reduction of Animals in Research, London, UK, ^2^School of Veterinary Science, University of Bristol, Bristol, UK, ^3^School of Biological Sciences, University of Bristol, Bristol, UK, ^4^National Heart and Lung Institute, Imperial College London, UK, ^5^Centre for Statistics in Medicine, University of Oxford, Oxford, UK.*

|  | | ITEM | RECOMMENDATION | Section/ Paragraph |
| --- | --- | --- | --- | --- |
| 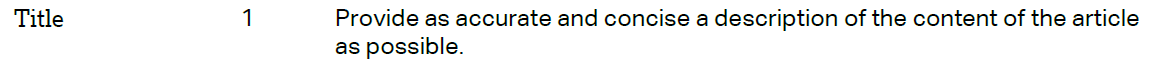 | | | Title Page  Page 1 |  |
| 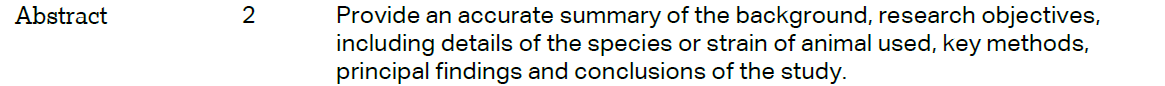 | | | Page 2 |  |
| INTRODUCTION | | |  |  |
| 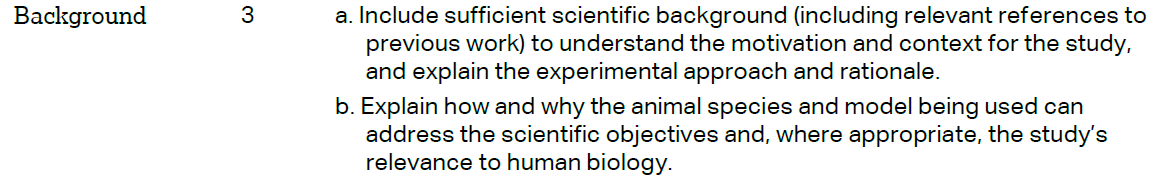 | | | Page 3-5 |  |
| 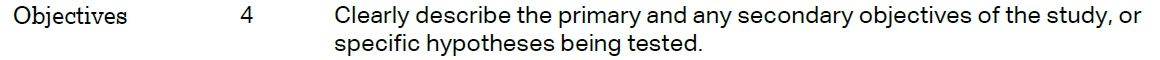 | | | Page 5  Line 7 |  |
| METHODS | | |  |  |
| 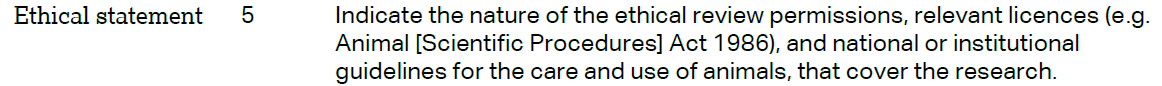 | | | Page 6  Line 2 |  |
| 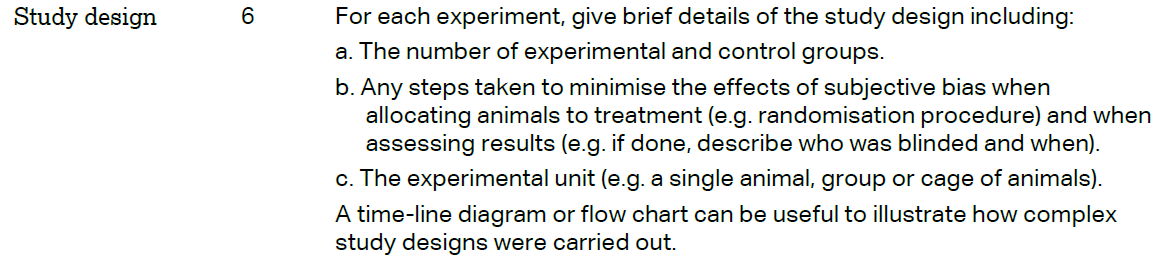 | | | Page 6,7  Line 10 |  |
| 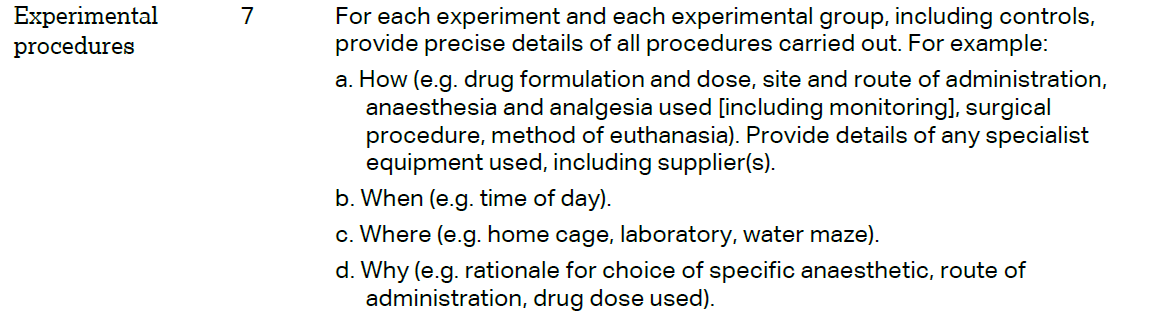 | | | Page 8 |  |
| 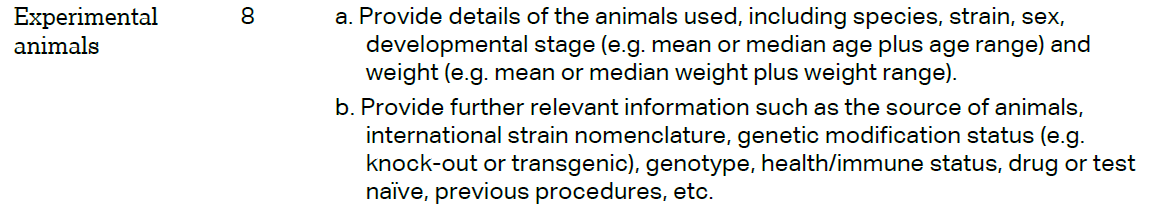 | | | Page 6,7 |  |

The ARRIVE guidelines. Originally published in *PLoS Biology*, June 2010^1^

| 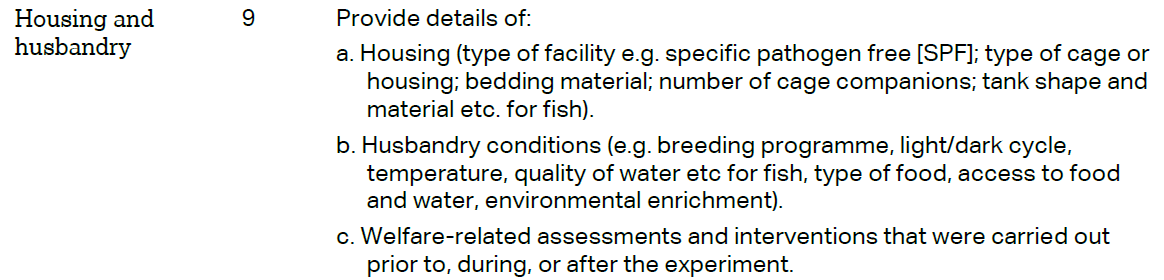 | Page 9 | |
| --- | --- | --- |
| 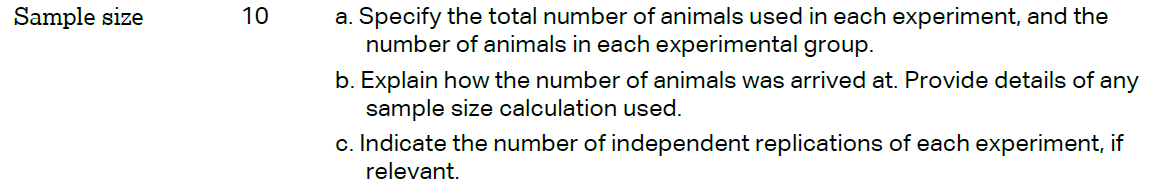 | Page 7 | |
| 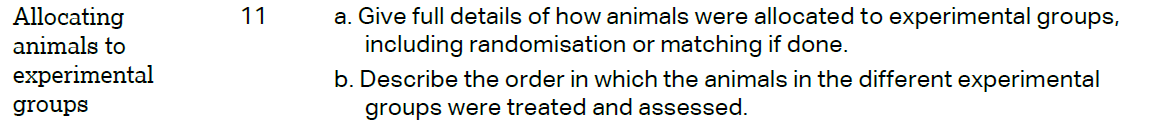 | Page 7 | |
| 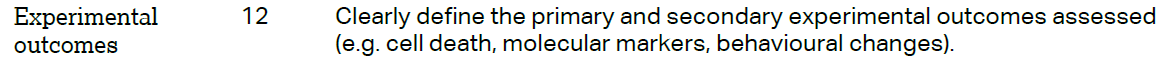 | Page 11 | |
| 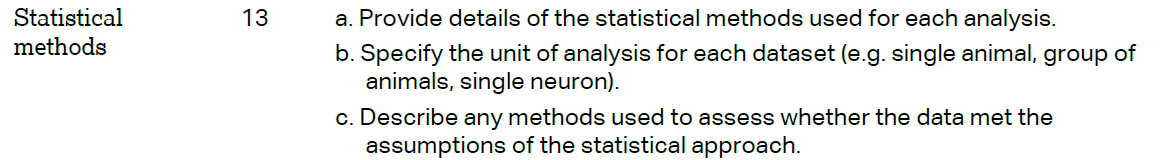 | Page 11  Line 18 | |
| RESULTS |  | |
| 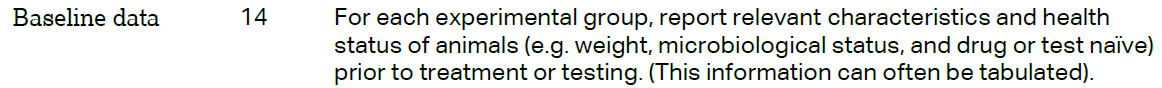 | Page 11 | |
| 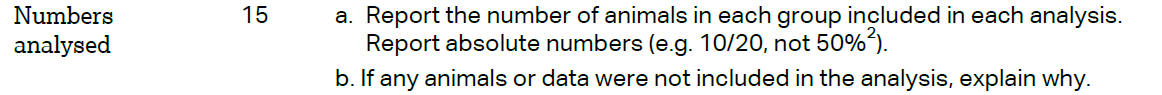 | Page 11 | |
| 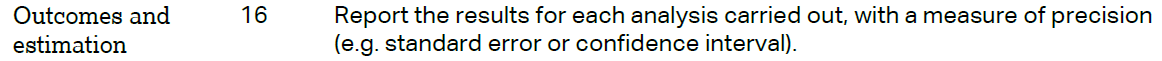 | Page 12-16 | |
| 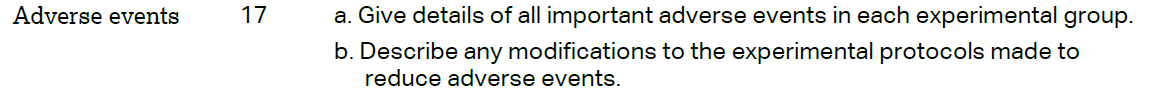 | Page 11 | |
| DISCUSSION |  | |
| 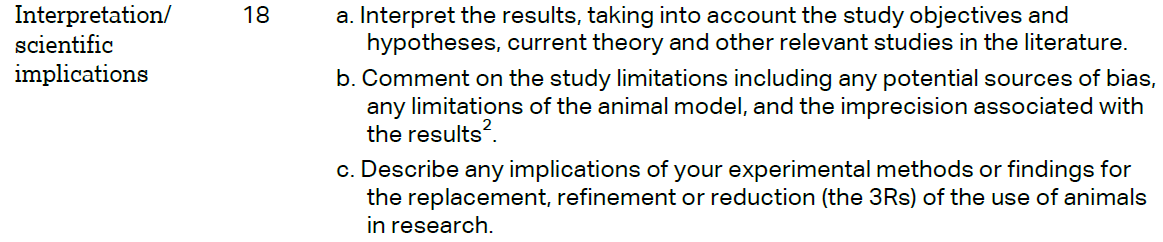 | Page 17-21 | |
| 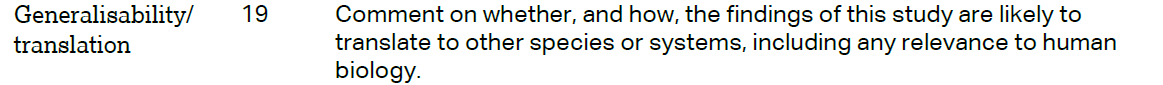 | Page 20,21 | |
| 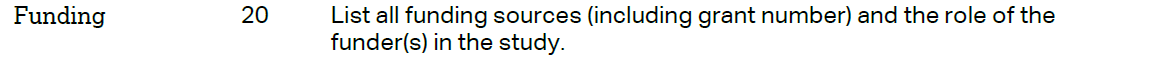 | | Title page,  Page 21 |


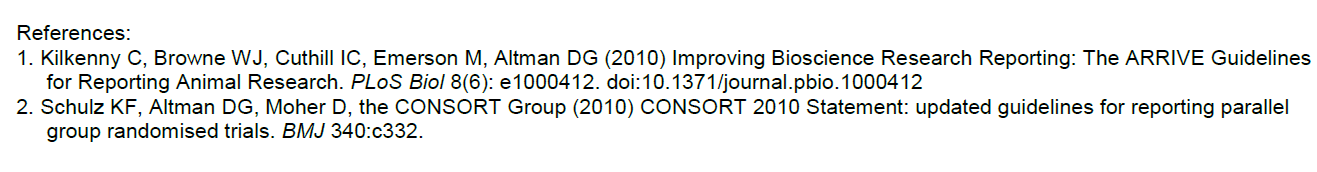

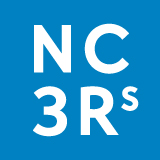

Supplement: S1 Checklist — (DOCX) [file pone.0202957.s001.docx]
